# Individual and Relationship-Level Correlates of Transactional Sex Among Adolescent Girls and Young Women in Malawi: A Multilevel Analysis

**DOI:** 10.1007/s10461-021-03442-2

**Published:** 2021-08-23

**Authors:** Margaret W. Gichane, Nora E. Rosenberg, Catherine Zimmer, Audrey E. Pettifor, Suzanne Maman, Bertha Maseko, Kathryn E. Moracco

**Affiliations:** 1 Department of Health Behavior, Gillings School of Public Health, University of North Carolina at Chapel Hill, Chapel Hill, NC USA; 2 Present Address: Department of Obstetrics, Gynecology & Reproductive Sciences, Advancing New Standards in Reproductive Health, University of California, San Francisco, Oakland, CA USA; 3 UNC Project, University of North Carolina at Chapel Hill, Lilongwe, Malawi; 4 HW Odum Institute for Research in Social Science, University of North Carolina at Chapel Hill, Chapel Hill, NC USA; 5 Department of Epidemiology, Gillings School of Public Health, University of North Carolina at Chapel Hill, Chapel Hill, NC USA

**Keywords:** Transactional sex, Adolescent girls, Young women, Partners, Multilevel

## Abstract

Transactional sex increases HIV risk among adolescent girls and young women (AGYW). Understanding the individual and dyadic nature of transactional sex may provide evidence for risk reduction interventions. Multilevel logistic regression was used to cross-sectionally examine correlates of transactional sex among AGYW in Lilongwe, Malawi. Participants (N = 920) reported 1227 relationships. Individual-level associations were found between being divorced/widowed (AOR 5.07, 95% CI 1.93, 13.25), married (AOR 0.26, 95% CI 0.09, 0.72), or unstably housed (AOR 7.11, 95% CI 2.74, 18.47) and transactional sex. At the relationship-level, transactional sex occurred in relationships with: non-primary primary partners (AOR 4.06, 95% CI 2.37, 6.94), perceived partner concurrency (AOR 1.85, 95% CI 1.11, 3.08), and feared violence with couples HIV testing (AOR 2.81, 95% CI 1.26, 6.29), and less likely to occur in relationships with children (AOR 0.15, 95% CI 0.06, 0.38). Multiple co-occurring social and structural vulnerabilities increase transactional sex engagement warranting the need for social protection and gender transformative approaches.

## Introduction

Adolescent girls and young women (AGYW) in sub-Saharan Africa (SSA) are a population at increased risk of HIV infection. AGYW ages 15–24 years old account for a quarter of new HIV infections in the region [1]. Transactional sex, defined as, “noncommercial, nonmarital sexual relationships motivated by an implicit assumption that sex will be exchanged for material support or other benefits [2, 3],” increases AGYW’s risk of acquiring HIV [4, 5]. Understanding the nature of transactional sex may allow us to reduce the risk within these relationships, which may in turn have an impact on HIV transmission rates among AGYW.

Studies of transactional sex in Malawi show the frequency and complexity of the behavior across a variety of settings [6–9]. Male provision of gifts to their female partners is integral role to the quality and duration of relationships of young rural Malawians. Gifts and money symbolize trust, demonstrate love, confer social status, and display gendered expectations of males as providers. Within relationships, the value of the item a girl receives may indicate her value to her partner. Further, couples report negotiating exchanges to retain their relationship [6]. Youth living in urban slums in Blantyre describe transactional sex as a common practice that is a product of material depravation that young people face in their communities. In particular, transactional sex is seen as a method to acquire money or goods in response to, unstable or poor housing conditions, food insecurity, and limited access to medical treatment. However, for young women, transactional sex is also seen as a tool that they can use to their advantage in different situations, and that can be used to acquire consumer goods [8]. Most evidence about exchange in transactional relationships comes from qualitative studies, which are limited in their generalizability. Systematically quantifying who is engaged in transactional relationships, what is exchanged, and distinguishing these features from non-transactional relationships, may provide critical insights about AGYW’s motivations to engage in these relationships and ultimately make them safer.

To date, studies of transactional sex have primarily focused on the characteristics of AGYW who engage in the behavior, ignoring characteristics of the relationships. Socioeconomic characteristics such as food insecurity [10, 11–13], housing [10, 11], orphan status [14, 15], education [10, 11, 13], and household wealth [11, 12] increase AGYW’s likelihood of engaging in transactional sex. Among the studies that have examined partner characteristics, age differences between partners have received the most attention [16–18]. Relationships in which there is a substantial age-difference (≥ 5 years or ≥ 10 years) between partners are often transactional [16–18]. Additionally, relationships which are characterized by partner concurrency [18] or violence [19], as well as those which are more casual [20, 21], are more likely be transactional.

Examining individual and relationship characteristics concurrently may lead to more comprehensive insights surrounding transactional sex, and potential interventions to make transactional relationships safer for AGYW. Multilevel modeling (MLM) is a rigorous analytical approach well suited to examine how factors at both the individual and relationship level are associated with a particular outcome [22]. MLM has been used widely to examine how individual demographics and partner traits predict risky encounter-level sexual behaviors such as condom use [23, 24]. MLM accounts for the hierarchal structure of data which permits the examination of multiple partnerships within the same person. One of the benefits of this approach is that it can assess whether individual-level characteristics (e.g., food insecurity, housing) and relationship characteristics (e.g. age difference, partner type) are robust predictors of behaviors across partnerships [25].

The purpose of this study is to characterize AGYW’s transactional sexual relationships. Specifically, we seek to: (1) describe and compare the gifts and material support received from transactional and non-transactional partners; and (2) examine the separate and combined associations between individual-level and relationship-level characteristics, and transactional sex engagement.

## Methods

### Study Design and Population

Data for this analysis were from the baseline assessment of Girl Power-Malawi, a quasi-experimental prospective cohort study conducted in four public-sector clinics Lilongwe, Malawi from February 2016-August 2017. Girl Power-Malawi Participants were recruited and enrolled in one of four models of care including: the standard of care, youth friendly health services (YFHS), YFHS and a 12 session group-based behavioral intervention, and the former along with a conditional cash transfer (CCT) based on attendance to behavioral intervention (BI) sessions. More detailed information about the study design and main findings are published elsewhere [26–28].

Two-hundred and fifty participants were recruited from each of the four clinics via community outreach activities, chain-referral from current study participants, and self-referral (N = 1000 total). Eligibility criteria included: age in the 15–24-year range, residence in proximity to the study clinic, and willingness to enroll in the study for a 1-year period.

Interviews were conducted in Chichewa by trained female research assistants. Data were captured using Open Data Kit (ODK) software on Android tablets. A baseline behavioral survey covered participant’s demographic information, sexual and reproductive behavior, and healthcare-seeking patterns. Participants were also asked to report information on their recent sexual partners in a partner grid. For each partner, participants were asked to report on characteristics of the relationship.

The following analyses use baseline behavioral survey to examine cross-sectional relationships between individual and relationship-level characteristics and transactional sex engagement. To examine these relationships, a relationship dataset was created with each individual line representing a different partner from the partner grid. Each participant could contribute information on up to three of their most recent relationships. Analysis was restricted to relationships which occurred in the last 6 months, thus, participants with no self-reported partnerships within the 6 months preceding baseline were excluded. Time period was assessed based on self-reported number of months since last intercourse with the partner.

### Measures

#### Descriptive Variables

Type and magnitude of material and monetary support were assessed for all partners. For material support, participants provided information on whether they received any of the following nine items: airtime, cell phone or other electronics, food, clothes or shoes, cosmetics (perfume, lotions, make-up), beauty treatments (hair, nails, braiding, etc.), music or videos, alcohol, and drugs. For material support, participants were asked how much money they received from partners and the frequency in which they received it.

#### Dependent Variables

The primary outcome of interest, “transactional sex,” was measured at the relationship-level. Transactional sex was a dichotomous outcome assessed by combining responses to two different measures. The first, was “Have you ever felt like you had to have sex with this partner because he gave you money or other things?” Response options were “yes,” “no,” and “do not know.” The second was, “What is the main reason you are with this partner?” Response options included: (1) looks/physical attraction; (2) love/romance/emotional support; (3) money/gifts/financial support; (4) adventure/entertainment; (5) status among friends; (6) family pressure; (7): do not know. Relationships were considered transactional if there was an affirmative response to the first question or if “money/gifts/financial support” was selected as the primary motivator in the second question. All marital relationships were considered non-transactional even if either two items designated the partner as transactional based on recent definitions of transactional sex [3, 29].

#### Independent Variables

The independent variables of interest were measured at the participant level as well as the relationship level. Participant level socioeconomic status (SES) variables refer to demographic information and the resources a participant or their household possess. The SES factors examined included: marital status, educational attainment, orphan status, food insecurity, unstable housing, savings, employment, running water, floor type, electricity, and asset index. Relationship level factors pertain to the characteristics of the partnership. The relationship-level factors include the age difference between the participant and partner, primary or non-primary relationship, marital relationship with participant, cohabitation with the participant, children with the participant, Whether the female participant perceived that her partner had concurrent partners during the past 6 months, ever tested for HIV as a couple with the participant, fear violence if tested with the participant, and fear abandonment if tested with the participant. All relationship-level variables were reported by the female participant.

### Covariates

Participant age was included a priori as a covariate as it is a potential confounder of the relationship between SES and sexual behavior. Also, given that participants were recruited from four clinics, dummy variables for clinic were constructed to account for any differences attributed to the settings they were recruited from.

### Analyses

We began by reporting univariate socioeconomic characteristics of the sample using frequencies and percentages. We then used the relationship dataset to assess the characteristics of AGYW’s reported relationships. Next, we calculated material and monetary support associated with each relationship. For material support, we examined differences in the proportion of participants who reported receiving each of the nine gifts based on whether the partner was transactional or non-transactional using Pearson chi-squared tests. For monetary support, we calculated medians, IQRs, and two-sample t-tests comparing the monthly amount of money received from transactional versus non-transactional partners.

To examine individual and partner level factors associated with transactional sex, we used multilevel modeling (MLM) as an analytical approach. MLM was selected to account for the nesting of partners (Level 1) within each individual AGYW (Level 2). We ran bivariate and multivariable mixed effects logistic regression using Stata 15 (College Station, TX). We first fit separate bivariate mixed effects logistic regression models with each individual level and partner level factor and transactional sex. Next, we ran three multivariable mixed effects logistic regression models to examine the separate and combined relationships between individual and relationship-level factors and transactional sex. Model 1 included all individual level characteristics. Model 2 included all partner level characteristics. In Model 3, the final model, we entered all individual and partner level characteristics that were significant at p < 0.20 in the individual models and removed variables using a backward elimination procedure, retaining factors which were significant at p < 0.05. All three models controlled for age and clinic. All models used a logit link and binomial distribution to estimate odds ratios and 95% confidence intervals.

### Ethics

Prior to participation, consent was obtained from participants 18–24 years old, and assent and consent from a parent, guardian, or authorized adult were obtained for participants 15–17. The study was approved by the University of North Carolina Institutional Review Board and Malawi National Health Sciences Research Committee.

## Results

### Sample and Relationship Characteristics

One thousand participants were interviewed at baseline. Among them, 65 (7%) reported not having any sexual relationships within the past 6 months and 15 (2%) did not respond to key questions assessing transactional sex. Thus, the analyses included 920 AGYW (Table 1). The median age in the sample was 17 (IQR 15–21). The majority of participants were unmarried (70%). Twenty-three percent were married, and seven percent were divorced or widowed. Most had completed primary school (70%) and had two living parents (65%). Food insecurity was fairly common in this sample with over a third of participants reporting going to bed hungry on 1 or more days in the past month. A few AGYW reported being unstably housed (7%). Over half of participants (54%) were unemployed and the rest were enrolled as students (28%) or working for pay/self-employed (15%). Most lived in homes with no running water (58%) or electricity (63%). About one third (31%) had earth/sand floors in their homes and 40% had two of fewer assets.

**Table 1:** Demographic characteristics (N = 920)

	n	%
Age		
15–19	529	58%
20–24	321	35%
Marital status		
Single	646	70%
Married	209	23%
Divorced/widowed	64	7%
Missing	1	0%
Education level		
Did not complete primary	266	29%
Completed	646	70%
Missing	8	1%
Orphanhood		
Both parents alive	597	65%
Single orphan	244	27%
Double orphan	79	9%
Experienced any food insecurity		
No	606	66%
Yes	314	34%
Unstably housed		
No	854	93%
Yes	66	7%
Have savings		
No	821	89%
Yes	96	10%
Missing	3	0%
Employment		
Unemployed/other	495	54%
Full-time student	256	28%
Working for pay/self-employed	160	17%
Missing	9	1%
*Household socioeconomic factors*		
Running water in home		
No	529	58%
Yes	391	43%
Housing material		
Earth or sand	284	31%
Cement/tile/other	635	69%
Missing	1	0%
Electricity in home		
No	577	63%
Yes	341	37%
Missing	2	0%
Asset Index		
≤ 2 assets	367	40%
≥ 3 assets	553	60%

These 920 participants reported a total of 1247 relationships in the past 6 months. Of these 920 participants, 74%reported one partner, 18% reported two partners, and 8% reported three or more partners. Outcome data were missing for 20 relationships, making the total partner sample 1227 (Table 2). The age difference in the majority of relationships was less than 10 years (89%) and most were primary relationships (78%) (Table 2). Of reported relationships, few were marriages (16%), or included children together (19%) or cohabitation (15%). The majority (55%) of relationships had lasted longer than 6 months. Twenty-six percent of partners had been tested for HIV with the participant as a couple. Fear of physical violence or abandonment prevented AGYW from seeking couple’s HIV testing with 8% and 12% of partners, respectively.

**Table 2:** Relationship characteristics and sexual risk factors (n = 1227)

	n	%
*Relationship characteristics*		
Intergenerational		
< 10 years age difference	1097	89%
≥ 10 years older	57	5%
Unknown partner age	73	6%
Relationship type		
Primary	958	78%
Non-primary	267	22%
Children together		
No	990	81%
Yes	234	19%
Missing	3	0%
Cohabitate		
No	1038	85%
Yes	189	15%
Sexual concurrency		
No	581	47%
Yes/don't Know	643	52%
Missing	3	0%
Relationship length		
1 day	161	13%
< 6 months	378	31%
≥ 6 months	675	55%
Missing	13	1%
Couples HIV testing		
Have not been tested as couple	906	74%
Have been tested as a couple	316	26%
Missing	5	0%
Fear physical violence if tested together		
No/have tested as a couple	1106	90%
Yes	94	8%
Missing	27	2%
Fear abandonment if tested together		
No/have tested as a couple	1039	85%
Yes	153	12%
Missing	35	3%

Twenty-two percent of relationships were transactional.

### Material and Monetary Support

**Fig. 1:**
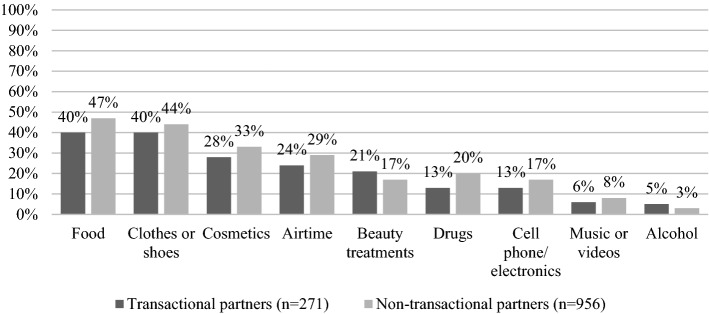
Items received from transactional vs. non-transactional partners

AGYW were more likely to receive gifts from non-transactional versus transactional partners (61% vs. 55%, p = 0.042). The top three items received from partners was the same across partner type and included food, clothes or shoes, and cosmetics (Fig. 1). Out of the nine items listed, AGYW received an average of 1.9 items (SD 2.4) from transactional partners and 2.2 items (SD 2.4) from non-transactional partners.

In contrast, more transactional partners (96%) compared to non-transactional partners (85%) provided money (p < 0.001). Of the 1074 partnerships where money was provided, transactional partners provided more money compared to non-transactional partners. The median amount provided was $8.28 (IQR $4.14–16.55) from transactional partners and $6.90 (IQR $2.76–13.79) for non-transactional partners. T-tests comparing the mean amount provided by partners, indicated that AGYW tend to receive more from transactional partners (M = $15.85, SD $20.05), compared to non-transactional partners (M = $13.50, SD $21.19), though findings were marginally significant (p = 0.06).

### Individual-Level Factors Associated with Transactional Sex

Several individual level factors were associated with transactional sex. In bivariate analyses, being divorced, being a double orphan, being unstably housed, living in a home without running water, and having few household assets were associated with transactional sex. After adjusting for all individual level factors, age, and clinic, there was some attenuation among factors associated with transactional sex (Table 3, Model 1). Being divorced or widowed (AOR 4.94, 95% CI 1.85, 13.19), married (AOR 0.08, 95% CI 0.03, 0.23), a double orphan (AOR 1.86; 95% CI 0.73, 4.76), unstably housed (AOR 4.65, 95% CI 1.80, 11.99), unemployed (AOR 2.26, 95% CI 0.95, 5.42), as well as having no savings (AOR 0.52, 95% CI 0.22, 1.24), or living in a home with earth/sand flooring (AOR 0.50, 95% CI 0.23, 0.88), and having fewer assets (AOR 1.59, 95% CI 0.81, 3.13) met the cut-off point of < 0.20 for inclusion in model three.

**Table 3:** Multilevel logistic regression of individual and partner level correlates of transactional sex

Individual	Model 1: individual		Model 2: partner		Model 3: combined	
	AOR	95% CI	AOR	95% CI	AOR	95% CI
*Individual Socioeconomic factors*						
Marital status (ref = single)						
Single	Ref				Ref	
Married	0.08***	(0.03, 0.23)			0.26**	(0.09, 0.72)
Divorced/widowed	4.94**	(1.85, 13.19)			5.07**	(1.93, 13.25)
Educational attainment						
Primary school or higher	Ref					
Less than primary school	1.16	(0.60, 2.28)			–	–
Orphanhood						
Both living parents						
Single orphan	1.32	(0.72, 2.41)			–	–
Double orphan	1.86	(0.73, 4.76)			–	–
Food insecure						
No	Ref					
Yes	1.00	(0.93, 1.06)				
Unstable housing						
No	Ref				Ref	
Yes	4.65**	(1.80, 11.99)			7.11***	(2.74, 18.47)
Savings						
Have savings	Ref					
No savings	0.52	(0.22, 1.24)			–	–
Employment						
Employed	Ref					
Full-time student	1.73	(0.63, 4.73)			–	–
Unemployed	2.26	(0.95, 5.42)			–	–
*Household Socioeconomic Factors*						
Running water						
Running water	Ref					
No running water	1.21	(0.58, 2.52)			–	–
Flooring						
Cement/tile	Ref					
Earth/sand	0.50*	(0.23, 0.88)			–	–
Electricity						
Electricity in home	Ref					
No electricity	1.21	(0.60, 2.45)			–	–
Assets						
≥ 3 assets	Ref					
≤ 2 assets	1.59	(0.81, 3.13)			–	–

Partner level						
*Relationship characteristics*						
Age difference						
< 10 years			Ref			
≥ 10 years older			3.28*	(1.07, 10.06)	–	–
Unknown partner age			1.08	(0.36, 3.20)	–	–
Relationship type						
Primary			Ref		Ref	
Non-primary			4.42***	(2.48, 7.88)	4.06***	(2.37, 6.94)
Children together						
No			Ref		Ref	
Yes			0.12***	(0.05, 0.31)	0.15***	(0.06, 0.38)
Partner concurrency						
No other partner			Ref		Ref	
Has other partners/unknown			1.73*	(1.02, 2.94)	1.85*	(1.11, 3.08)
Relationship length						
≥ 6 months			Ref		–	–
1 day			1.14	(0.52, 2.48)	–	–
< 6 months			1.82*	(1.02, 3.26)	–	–
Couples HIV testing						
Tested together			Ref			
Not tested together			1.04	(0.55, 1.98)	–	–
Fear physical violence if tested together						
No/have tested as a couple			Ref		Ref	
Yes			2.23	(0.75, 6.60)	2.81*	(1.26, 6.29)
Fear abandonment if tested together						
No/have tested as a couple			Ref			
Yes			1.13	(0.45, 2.84)	–	–

Adjusted for age and clinic

*p < .05, **p < .01, ***p < .001

### Relationship-Level Factors Associated with Transactional Sex

Transactional sex was more common with partners with certain characteristics. In bivariate models, odds of transactional sex were higher in the following relationships: ≥ 10-year age difference, non-primary, shared children with partner, perceived to be in concurrent relationships, and short-term relationships (< 6 months and 1 day). Additionally, transactional sex was more common in relationships in which they had not received couple’s HIV testing, and in those they feared physical violence or abandonment if they were to test together. Similar to Model 1, after adjusting for all relationship-level variables, age, and clinic, the effect sizes of variables found to be significant in bivariate analyses were attenuated (Model 2). Odds of transactional sex remained higher in relationships with partners who were: ≥ 10 years older (AOR 3.28, 95% CI 1.07, 10.06), non-primary (AOR 4.42, 95% CI 2.48, 7.88), < 6 months long (AOR 1.82, 95% CI 1.02, 3.26), and those in which the female participant believed were in concurrent relationships (AOR 1.73, 95% CI 1.02, 2.94) or they feared physical violence if they tested for HIV together (AOR 2.23, 95% CI 0.75, 6.60).

### Combined Individual and Relationship-Level Factors Associated with Transactional Sex

The final model examined the combined effects of individual and relationship-level factors. Among individual-level factors, being divorced/widowed (AOR 5.07, 95% CI 1.93, 13.25) and being unstably housed (AOR 7.11, 95% CI 2.74, 18.47) were associated with increased odds of transactional sex. Being married was associated with reduced odds of transactional sex (AOR 0.26, 95% CI 0.09, 0.72). At the relationship-level, non-primary relationship type (AOR 4.06, 95% CI 2.37, 6.94), perceived partner concurrency (AOR 1.85, 95% CI 1.11, 3.08), and fear of violence with testing (AOR 2.81, 95% CI 1.26, 6.29), were associated with elevated odds of transactional sex. Having a child with the partner was protective against transactional sex (AOR 0.15, 95% CI 0.06, 0.38).

## Discussion

This study provides an in-depth analysis of transactional sexual relationships among AGYW in Malawi. We found that AGYW receive similar types of items from transactional versus non-transactional partners, however, they receive more money from transactional partners. In multivariable models, we found that at the individual-level being divorced or widowed, married, or unstably housed were robust correlates of transactional sex; at the relationship-level, AGYW had increased odds of transactional sex in relationships with: perceived partner concurrency, non-primary relationship status, and feared violence with couples HIV testing.

AGYW in the study received similar items and amounts of money from transactional versus non-transactional partners. We found that participants were primarily receiving lower valued items, however monetary values were quite high in contrast to general incomes in Malawi ($27 per month) [30]. These findings align with previous research in Malawi highlighting the normative practice of gift giving and monetary support in adolescent and young adult relationships [31, 6], and provides nuance by examining the range and value of what is provided. Our research contributes to a growing body of work exploring the value of exchanges in transactional partnerships [32–34]. Partnerships in which higher valued items are exchanged are viewed as riskier as they may put young women in situations where they may feel unable to refuse sex or negotiate condom use [35, 36].

AGYW who were divorced or widowed or unstably housed had high odds of engaging in transactional sex. Although these groups were a small subset of the sample (7% divorce or widowed, and 7% unstably housed), they represent a population facing increased instability. Homelessness and unstable housing [37] have been previously linked to transactional sex among young adult populations, and in Malawi in particular [38], but there is limited exploration of the link between divorce/widowhood and transactional sex [39]. With limited opportunities for formal employment, AGYW who lose a long-term partner may be forced to look for economic support from transactional partners.

The findings on partner characteristics most strongly associated with transactional sex are consistent with previous work on this topic. Partner age-difference was highly associated with transactional sex, which is consistent with studies examining the characteristics of men reporting engaging in transactional sex with AGYW [17, 40, 41]. However, it is important to note that few relationships were confirmed to be with a partner 10 or more years older (intergenerational relationships) (5%), though some of the relationships in which the partner age was unknown (6%) may have been with a partner 10 or more years older. Furthermore, intergenerational relationships only accounted for 11% of all transactional sexual relationships that were recorded, which supports research refuting the notion that “sugar daddies” (older men who date younger women and provide large sums of money) are the primary type of transactional relationship [17]. While less common, transactional relationships with older partners may be among the riskiest because HIV prevalence among men 25–29 (6.4%) and 30–34 (9.2%) in Malawi, is much higher than that of men of similar age to AGYW in the study (1% among 15–24 year old men) [42].

Participants reported that they knew or suspected that over half of partners had other concurrent partners, and transactional sex was more common in these types of relationships. These findings corroborate evidence citing high levels of concurrency among men in Eastern and Southern Africa [18, 43]. This is of particular concern as concurrency heightens AGYW’s exposure to high risk sexual networks [18]. A study among men in South Africa found that men’s non-primary partners tend to be younger than primary partners [18]. Given that HIV prevalence increases with age in Malawi [42], having partners whose concurrent partners are in older age groups can elevate AGYW’s potential for acquiring HIV.

Transactional sex was more commonly reported in relationships in which AGYW feared their partner would be physically violent if they suggested couple’s HIV testing. This finding is concerning for two reasons: (1) if AGYW fear violence resulting from HIV testing, then it is likely that they may be unable to negotiate other protective sexual behaviors with their partner; and (2) if the partner is unwilling to be tested as a couple, there is lower likelihood of mutual disclosure of HIV status in the relationship. Couples HIV testing is more protective against HIV compared to individual HIV testing because it increases condom use among serodiscordant couples [44]. Couples HIV testing may not be the most appropriate intervention for these types of relationships.

Our findings indicate that AGYW’s social and structural vulnerabilities increase the likelihood of engagement in transactional sex in this setting. Structural approaches which have had some success in reducing transactional sex engagement include government cash transfers [45], providing tuition and uniforms to keep girls in school [46], and combination social protection approaches which combine cash transfers, free schooling, and parental monitoring [47]. In South Africa, the integration of government cash transfers, free schooling, and parental monitoring was associated with a 9% reduction in transactional sex engagement among AGYW ages 12–18 [47]. However, it is possible that current effective structural approaches may miss some of the most vulnerable AGYW we identified through this research. AGYW who are divorced or widowed and those who are unstably housed may need additional supports including support in acquiring housing, larger cash transfers or vocational training. Research is needed to adapt current effective approaches to meet the unique needs of these vulnerable subpopulations.

Additionally, while efforts to address transactional sex have primarily focused on reducing structural vulnerabilities of young women, there is a critical need for approaches which address the behavior of transactional male partners. Gender transformative interventions which seek to change norms around sexual concurrency, intergenerational relationships, and HIV testing may help reduce women’s vulnerability in transactional relationships [48]. Examples of such interventions include Stepping Stones and SASA which have shown reductions in key behaviors including, perpetration of intimate partner violence, transactional sex, condomless sex, and partner concurrency [49, 50].

There are a few limitations in this study. First, the cross-sectional study design restricts our ability to infer causal relationships. However, these findings corroborate longitudinal evidence from this same cohort showing similar relationships between SES and transactional sex [51]. Despite the cross-sectional design, this study provides novel information about the role of individual and relationship characteristics in transactional sex engagement. The addition of relationship level factors highlights how transactional sex is shaped both by the characteristics of young women and the partners they are with. Second, there is the potential for information bias. Partner characteristics and behavior were self-reported and subject to recall bias which may have affected associations with transactional sex. To address recall bias, our analyses only focused on relationships within the past 6 months to ensure the most accurate capture of partner characteristics. Third, important factors which may impact engagement in transactional sex, such as family context [52], were not included in analyses. Fourth, the monetary and material support from partners may not be comprehensive of all support provided, and thus may obscure possible differences between transactional and non-transactional partners. Finally, this study sample was not recruited using nationally representative methods, thus findings may not be generalizable to all AGYW in Malawi.

### Conclusions

AGYW vulnerability to acquiring HIV is determined, in large part, by their social and structural environment. Current research has primarily focused on the traits of AGYW, ignoring the relationship context. Our work shows that AGYW’s individual socioeconomic circumstances lead to engagement in transactional relationships characterized by HIV risk. Social protection approaches which address multiple dimensions of SES are urgently needed for HIV prevention for this population.

## Data Availability

Not Applicable.
